# Progressive Age-Associated Blood–Brain Barrier Leak/Dysfunction-Nexus of Neurodegenerative Disease Using MRI Markers to Identify Preclinical Disease and Potential New Targets for Future Treatments

**DOI:** 10.3390/diagnostics14070726

**Published:** 2024-03-29

**Authors:** Charles R. Joseph

**Affiliations:** Neurology and Internal Medicine, College of Osteopathic Medicine, Liberty University, Lynchburg, VA 24502, USA; crjoseph@liberty.edu

**Keywords:** blood–brain barrier, microvascular pathology, neurodegenerative inflammatory changes, ASL MRI, DCE MRI, serologic markers

## Abstract

This review article focuses on the upstream pertinent pathophysiology leading to neurodegenerative disease. Specifically, the nexus appears to be blood–brain barrier (BBB) leakiness resulting in a two-prong inflammatory disease spectrum damaging the microvasculature and corrupting protein synthesis and degradation with accumulating misfolded toxic proteins. The suboptimal results of removing misfolded proteins mean a new approach to disease in the preclinical state is required aimed at other targets. Validated noninvasive imaging and serologic biomarkers of early preclinical disease implemented in the high-risk patient cohort along with periodic surveillance once effective treatments are developed will be required. This review discusses the physiology and pathophysiology of the BBB, new MRI imaging techniques identifying the leak, and altered fluid dynamic effects in the preclinical state. The risk factors for disease development, preventative measures, and potential treatment targets are also discussed.

## 1. Introduction

Throughout the past few years, the pharmacologic focus on the removal of β amyloid has proven disappointing [1,2]. Their accumulation occurs later and perhaps reaches a non-recoverable point in the neurodegenerative process. The early inflammatory phase begins years or decades prior to their accumulation in association with microvascular disease [3,4,5,6,7]. It has become clear that the nexus of this complex disease is blood–brain barrier dysfunction (BBB) [3,6,7,8]. The scope of this chapter includes a discussion of the normal physiology of the BBB, mechanisms of damage, the physiologic consequences of dysfunction and methods of early detection of vascular injury. What is known of the normal restorative function after BBB damage will be discussed in the context of apparent decline with aging. This will precede a discussion of the pathophysiology of BBB damage and its perpetuation and acceleration by accumulating toxic protein end products (β Amyloid and hp Tau) from corrupted synthesis and degradation. Evidence-based discussion of prevention and a focus on methods of noninvasive early detection and probable future therapeutic research directions follow.

Here-to-fore therapeutic efforts at mitigating Alzheimer’s disease have focused on late-disease-stage removal of toxic protein end products (β Amyloid) with limited effect and great expense [1,2]. It is clear from these clinical trials, however, that early intervention is necessary for success, perhaps in the preclinical state [9,10]. Further, a change in therapeutic target(s) addressing the upstream nexus (BBB dysfunction) leading to defined pathologic and clinical damage may suppress disease development. We will address what is known of early BBB pathophysiology, the multiplicity of potential triggers, and consequent altered fluid dynamics. The details of the various signaling pathways are not included for simplicity but several excellent review articles have been referenced for those interested [6,8,11].

Leveraging the early perfusion dynamic alterations, preclinical disease may be identified using invasive and noninvasive techniques to serve initially as an outcome measure of efficacy for new therapeutic endeavors [12,13,14,15]. They will potentially serve as both a diagnostic and surveillance tool to identify and assess outcomes of novel early-disease-phase treatments before cognitive decline develops.

### 1.1. Normal BBB Physiology and Regulation

**Figure 1:**
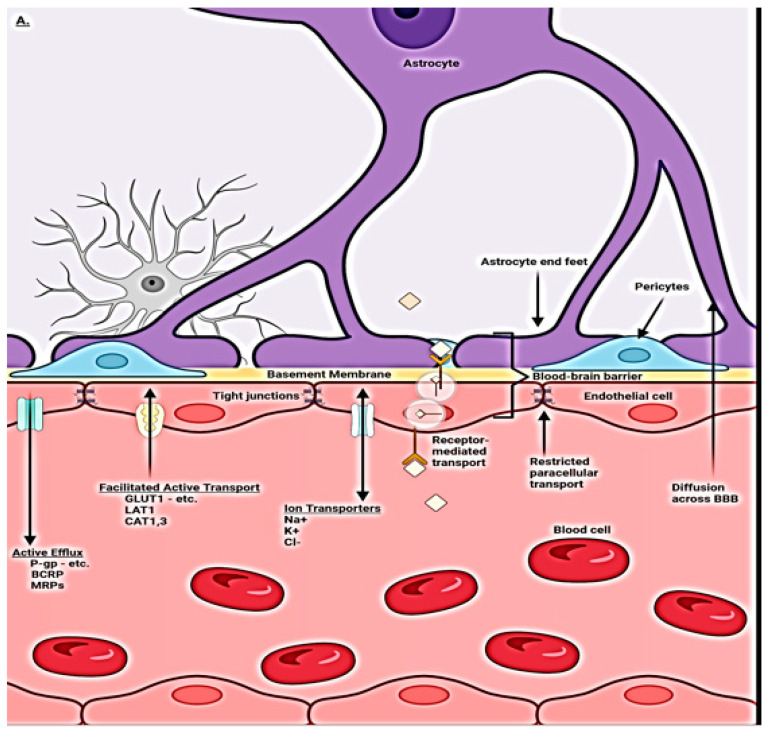

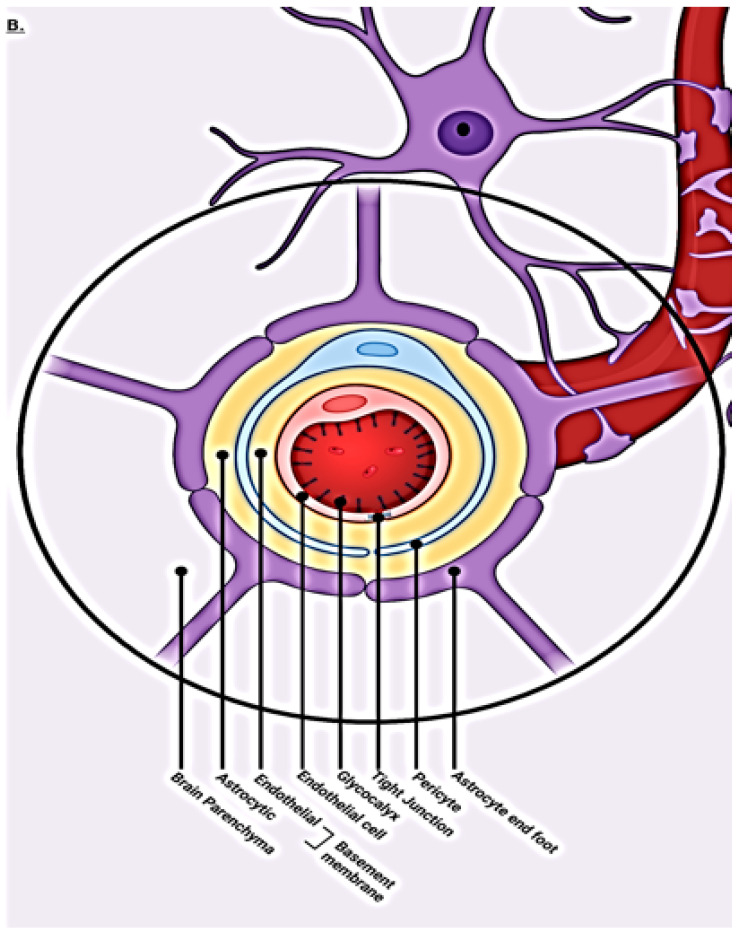
(**A**) Compendium of normal transport mechanisms through the BBB endothelium with NVU cellular relationships. The neurovascular unit/blood–brain barrier (NVU/BBB) is composed of specialized endothelial cells and support cells, including pericytes and astrocytes. The cross-sectional view illustrates that the majority of the abluminal surface of the endothelial cell is covered by pericytes and astrocytic foot processes. Paracellular transport across the BBB/NVU is restricted by tight junction proteins, and even small, lipophilic molecules. Facilitated active transport, receptor-mediated transport, and ion transporters allow the brain to be supplied with nutrients while maintaining strict homeostasis. Adapted from [21]. (**B**) Normal transaxial anatomy of the NVU/vascular (capillary) BBB. From the inside out, the layers that form the blood–brain barrier include the endothelial-expressed glycocalyx, specialized endothelial cells, double-layer endothelial- and astrocyte-expressed basement membrane with glymphatic space sandwiched between the layers, pericytes, and astrocytes. Adapted from [22].

The brain is a complex highly metabolically active organ requiring a steady flow of nutrients, oxygen, and selected substrates and waste removal for maximum efficient function. This requires highly regulated and unfettered rapid access to the above, depending on moment-to-moment need and, at the same time, excluding potentially damaging substances. The capillary vasculature provides both functions with unique highly specialized endothelial cells, which channel in nutrients passively and actively via ionic, nonionic, and small-molecule transporters as well as specialized vesicular clathrin pathways for selected larger molecules [3,8,11,16,17,18] (Figure 1A). Together with pericyte signaling, tight junctions (TJs) are formed between endothelial cells, which exclude potentially harmful molecular patterns, so-called damage-associated molecular patterns (DAMPS) from unwanted but innately produced substances and pathogen-associated molecular patterns (PAMPS) from foreign substances from infectious agents. It is the pericytes, via communication with glial cells of the neurovascular unit (NVU), that regulate uptake via BBB transport mechanisms of needed substrates. Waste byproducts, CO_2_, and water accumulating within the neuropil from oxidative metabolism are dealt with by both venous transport (≈60%) and ≈40% exits via the ingenious use of the paravascular space between the basement membranes of the pericytes and astrocyte end feet, dubbed the glymphatic space [19]. Counterintuitively, the waste fluid flow is retrograde through the intraarterial walls, ultimately admixing with CSF within the arterial Virchow Robin spaces and then moving outward to the subarachnoid space. Fluid is then reabsorbed through arachnoid granulations, the cribriform plate, and dural meningeal lymphatics and then ultimately drains into the deep cervical lymph nodes. There it rejoins peripheral lymphatic fluid flow returning to the blood circulation via the thoracic duct. A minor component leaves via sleeves of exiting spinal nerve roots [20]. As noted below, disruption of this pathway leads to the destructive accumulation of waste within the neuropil and arterial walls.

### 1.2. Blood–Brain Barrier Cellular Makeup and Their Function

**Figure 2:**
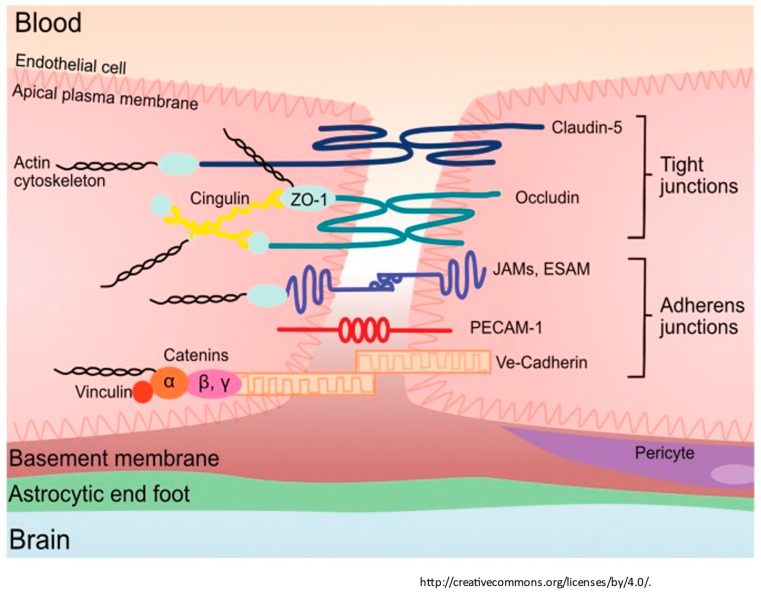
The tight junction proteins include claudin-5, occludin, and zonula occludins (ZO-1,2,3). Claudin-5 and occludin are both transmembrane proteins while the zonula occludens are intracellular proteins. The adherens junctions include transcellular components, JAMs, ESAM, PECAM-1, and Ve-cadeherin. The cytoplasmic catenins form a complex with Ve-cadeherin. Actin cytoskeleton helps to anchor the junctional proteins in endothelial cells. Adapted from [26].

The complex nature of the brain BBB requires interaction among all elements of the neurovascular unit (NVU) [5,8,9,16]. The high metabolic needs of the brain (8% of body mass but consumes 25% of produced energy) translate into the need for a steady inflow of nutrients and outflow of waste for optimal function. The system is highly dynamic in nature, adapting to rapid changes in metabolic requirements by expressing appropriate transporters and allowing for the selective ingress and egress of ions, lipids, and solutes, as well as transcytosis of selective larger molecules (Figure 2). O_2_ and CO_2_ move passively through the barrier. Maintenance of the tight junctions is also responsive to metabolic needs and highly selective at the same time, excluding potential toxins [23,24]. These functions are controlled by a choreography of signaling amongst all the NVU players (capillary endothelium, astrocytes, pericytes, and associated basement membranes). The exclusive nature of what passes through the barrier is controlled by layered protection. Starting from the intravascular space, negatively charged glycocalyx expressed by endothelial cells repels the entry of unwelcome plasma-associated proteins. The endothelial cells themselves express selective transporters and transcytosis pathways and the tight junctions between cells are expressed via direction from pericytes/astrocytes. Pericytes and astrocyte end feet and their basement membranes provide the final barrier [24,25] (Figure 1B). 

Autoregulation of the blood inflow to each region of the brain is controlled by smooth muscle cells (SMCs) in the precapillary arterioles and postcapillary venules with pericytes lining the capillaries [16,27]. SMCs at the arterial–capillary and capillary–vein junctions have cytoplasmic actin, which allows for the finetuning of pre and postcapillary flow, thereby maximizing oxygen and nutrient extraction [16,27]. Local energy requirements controlling blood flow are signaled by astrocytes to pericytes locally, which in turn optimize the vascular tone [28]. The autoregulatory vascular tone is balanced between local energy requirements and the mean arterial pressure (MAP). Vascular tone regulation is dependent on the rapidity of MAP changes and is more effective with MAP elevations than decreases. The dogma of stable CA over a wide range of MAP has been effectively disputed [29]. This latter issue is tangential to our discussion yet is critical in the clinical considerations of blood pressure management and its effect on cerebral perfusion. We will now address the function of the cellular components of the NVU in homeostasis and in disease. 

### 1.3. Capillary Endothelial Cells

The capillary endothelial cells (ECs) within the central nervous system take on enhanced roles in homeostasis, providing a gatekeeper function for the entry of nutrients and egress of waste [30,31,32]. The increased functionality of brain ECs relative to peripheral ECs requires additional energy needs and, thus, more mitochondria are present in the former [8,9,11]. The restrictive tight junctions consisting of intercellular protein strands are expressed by ECs [8,9,11]. Pericytes regulate the expression of these tight junctions and are, in effect, transducers of signals from the glial and neuronal NVU components [8,11]. The endothelial cells are responsible for the passive ingress of oxygen and the egress of CO2, lipid, and soluble molecules under four hundred Daltons or eight or fewer carbon bonds [8,11]. The active transport of ionic and nonionic (glucose and amino acids) small molecules and the transcytosis of selected larger molecules occurs via clathrin vesicular pathways [18] (Figure 1A).

The basement membrane matrix (BMM) of the capillaries is expressed on the abluminal side of and by ECs and pericytes. A second contiguous outer layer is expressed by astrocyte end feet. The BMM connects the endothelial cells firmly to the NVU [8,11]. Further, the BMM component and integrin protein ligands are a signal pathway from astrocytes to endothelial cells [26,33]. This intimate pathway signals the release of growth factors and receptors and influences cell survival, migration, and polarity [26,33].

On the luminal side of endothelial cells, glycocalyx is secreted, consisting of proteoglycans and heparan sulfate, which are negatively charged, thus repelling negatively charged plasma as well as blood cells from entry into the neuropil [34]. Further, heparan sulfates inhibit intravascular coagulation. In the presence of hypertension, the glycocalyx transduces sheer force energy, signaling the nitrous oxide synthetase to produce vasodilator nitrous oxide [34]. The subsequent effect is vasodilation, but in addition, ROS species are generated, causing the upregulation of metalloprotease enzymes with deleterious effects on tight junctions resulting in leaks [34]. The glycocalyx thus adds the first layer of protection for the BBB and its integrity affects the BBB stability [34].

### 1.4. Pericytes

Capillary endothelial cells are encased by pericytes, which regulate them. Pericytes express PDGF receptors with the cells migrating embiologically to endothelial cells, which express PDGF [7,8]. The two cell types become intimately connected via a ‘peg and socket’ arrangement, which allows for ease of signaling between them [11]. The ratio of endothelial cells to pericytes in the CNS is 1:1, demonstrating the importance of the relationship for continued maintenance of the TJ and the expression of transporters and transcytosis [7,8,11]. Their basement membrane and the BM of the surrounding astrocyte end feet serve as the conduit defined as the glymphatic space, which is thought to manage ≈40% of NVU metabolic waste egress [11]. In addition, they secrete important protein components incorporated in the tight junctions [11]. Pericytes also are necessary for aquaporin 4 channel expression in the astrocyte end feet tethered together by laminin proteins [35]. Further, pericytes express a variety of ionic and solute transporters, but their exact role is unknown [7,8,11]. Of importance, though, is their expression of Lipoprotein receptor LRP1, which facilitates the uptake and clearance of β amyloid. Unlike APOE 2 > 3 isoforms, which bind preferentially to LRP1 preventing the upregulation of CypA and upregulation of MMP-9, which if activated, degrades BBB tight junctions, APOE 4 does not [8,11]. The latter isoform allows for the accumulation of intracellular β amyloid in pericytes, which is toxic and results in their loss, disrupting the tight junctions (TJs) [8,36]. With damage to the BBB, pericytes are an early casualty, which causes a loss of polarization of the aquaporin channels (return to the soma from end feet). The loss of pericytes and Aquaporin channels in Astrocyte foot processes, plus leaking TJs, halts the glymphatic waste clearance pathway [7,8]. With the aging process, there is loss of capillary pericytes, but the mechanism is unknown [37,38,39]. 

### 1.5. Tight Junctions

Endothelial cells express cellular adhesion molecules and junctional proteins between endothelial cells, which function as a fine mesh and electrical barrier and effectively reduce the entry of unwanted small molecules and cells [23,24]. Starting on the luminal side of the endothelial cell junctions, Cadherin, PECAM 1, and other junctional proteins limit the ingress of leukocytes and allow for inter-endothelial cell signaling [23,24]. Transcellular junctional proteins of importance expressed by endothelial cells include transmembrane protein claudin 5 and occludins anchored to endothelial myosin filaments by intracellular Zona occludins, which restrict the paracellular ingress of ions and solutes [23,24]. The endothelial adhesion protein expression is upregulated by pericytes via signaling from angiopoietin release, thus inducing tight junction formation [8]. Astrocytes also express junctional proteins CX30/43, further strengthening the cell-to-cell adhesion [8]. The release of pro-maintenance retinoic acid, (SHH, and angiopoetin-1 enhances endothelial BBB maintenance [25]. In the presence of inflammation, microglia convert to proinflammatory Type 1 cells, which express C3a, which alters the phenotypic expression of endothelial cells, thereby retracting the tight junctions (see Figure 2) [8,37,38,39].

### 1.6. Astrocytes

Astrocytes also contribute to the BBB via their contribution in forming the outer basement membrane layer of glia limitans via the secretion of basement membrane proteins. This double layer of basement membrane serves as a second waste egress or glymphatic pathway [40]. The astrocyte end feet then surround the basement membrane and are thus involved in the ingress and egress of solutes, ions, lipids, and waste [40,41]. By monitoring and regulating neuronal metabolic activity, astrocytes signal the ECs’ required transporter expression for the import of needed substrates from the blood [8,40,41]. Water balance is also regulated via the end foot AQ 4 channels.

### 1.7. Microglia

The anti-inflammatory M2 microglia are homeostatic and protective of the BBB by providing anti-inflammatory cytokines, glucocorticoids, and matrix proteins. Their conversion to pro-inflammatory cells (type 1) when stimulated by either the ROS cascade or vascular or intrinsic borne cytokines upregulates the expression of inflammatory chemokines cytokines and complement release of the C3a fragment and subsequent phenotypic conversion of endothelial cells [42]. They convert ECs from a homeostatic phenotype to an, in effect, immune-attractant cell. As part of their conversion, the TJs are retracted, thus allowing inflammatory cells and substrates to enter the interstitium [43]. The effect of complement activation within the CNS is a clear contributor to accelerating continued BBB dysfunction and leak.

### 1.8. BBB Dysfunction

This extraordinarily complex barrier requires the full unfettered interaction of all the above-mentioned elements for the maximal protection and maintenance of brain function. The barrier likewise must have sustained resilience to constantly maintain function against the constant daily barrage of endogenous and exogenous insults. At the same time, with overwhelming infection or injury, the system must allow access to inflammatory cells and solutes [36]. The balance in youth is disrupted by traumatic brain injury (TBI), inflammation, or infection, but with resolution, there is spontaneous repair [44]. This balance begins to fail with the aging process, allowing a slow leak and a diminished repair capacity [38]. The pathways of restoration following low-level sustained or more significant injury are incompletely understood and clearly wane in advanced age [38,39]. Identified elements include age-related low-level inflammation or TBI with a reduction in pericytes and the upregulation of in situ inflammation resulting in leaky capillaries and microvascular anatomic disruption. Additionally, impaired glycocalyx protection and upregulation and circulation of inflammatory proteins systemically and within the neuropil enhance the loss of BBB integrity [45,46,47]. There is a consequent loss of pericyte homeostatic signaling, which results in reduced endothelial expression of junctional proteins and increased leakiness [38,39]. This provides a pathway for indiscriminate entry into the interstitium of inflammatory cells and normally restricted substances (fibrin, albumin, thrombin, hemoglobin, hemosiderin, immunoglobulins, free iron, and plasmin) [8,38,39]. Further, there is a loss of basement membrane integrity with consequent dysfunction of glymphatic flow and reduced communication, with the astrocyte-pericyte-endothelial signaling impairing the proper absorption of required solutes, ions lipids, and proteins [46,47,48]. Post-endothelial injury, these cells employ the Caveolae transcytosis pathway in lieu of the homeostatic Clathrin pathway, with the former being less discriminant in transporting appropriate proteins and lipids into the interstitium, thus adding more potential toxins [8,11]. Finally, astrocytes morph into a proinflammatory phenotype and pile on with the expression of Vascular endothelial growth factor (VEGF), matrix metalloprotease enzymes (MMPs), and endothelin, which add to the loss of BBB integrity and normal NVU signaling [48].

In youth, the WNT/βCatenin signaling pathway from astrocytes to endothelial structures enhances the expression and restoration of TJ proteins, restores transporter expression, and restores the homeostatic use of the clathrin transcytosis pathway [30]. The WNT/βCatenin pathway is enhanced by endothelial, pericyte, or astrocyte Netrin-activated endothelial Unc5b receptors [30]. Whether this is the only general restoration path restoring BBB integrity for all varieties of insults causing loss of BBB integrity is unknown.

**Figure 3:**
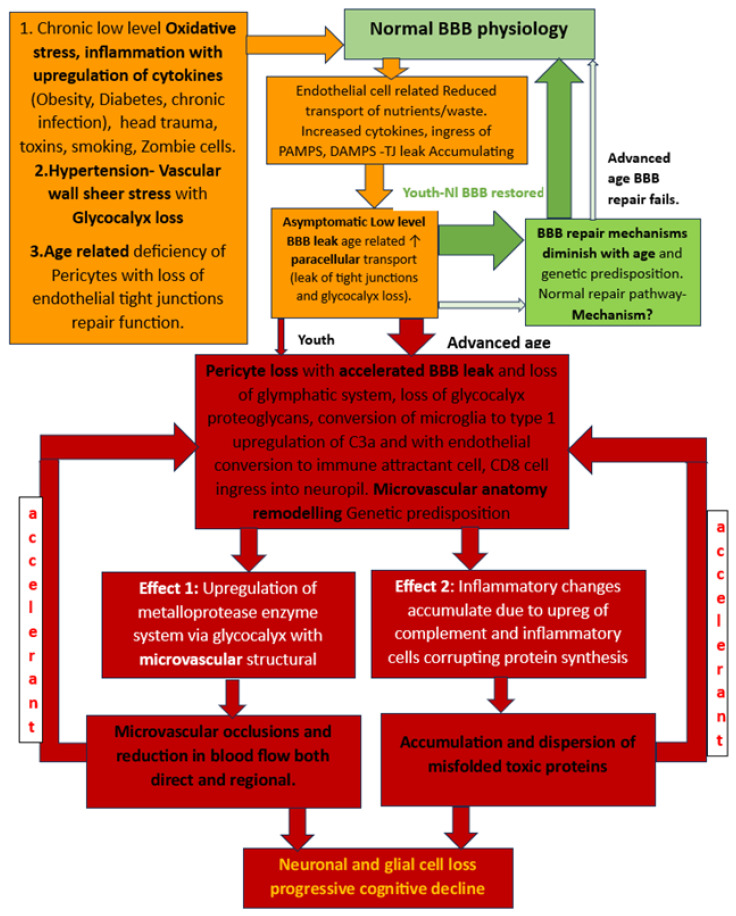
Flow chart of developing BBB dysfunction in youth with robust repair potential post injury and loss of repairability with advanced age and consequent worsening BBB damage and leak. The delayed result of the latter is 2-fold: microvascular damage and production and accumulation of misfolded proteins. The accelerant effect of accumulating β Amyloid and hpTau on further BBB disruption causes a viscous cycle of more widespread BBB dysfunction leading to more widespread vascular damage and inflammatory infiltration with the ultimate loss of neural elements and cognitive function decline.

In broad strokes, the main culprits affecting BBB integrity are blunt trauma, inflammation, viral or bacterial infection, and vascular injury. The causes are not mutually exclusive but are often co-occurring. The BBB targets of each of these insults vary, and as such, the initial downstream damage, whether it is vascular, metabolic, or both, may vary. We will explore each of the main culprits individually (Figure 3).

Head trauma causes biphasic injury, with the initial blunt force trauma initially disrupting normal BBB maintenance by its effect on the cellular and membranous components of the NVU [44]. Specifically, the loss of pericytes and their resulting effect on the loss of tight junction integrity and altered NVU signaling cause cerebral edema and leakage of solute and cellular components [44]. The second phase is inflammatory, which yields a double hit to the BBB. The influx of cytokines, inflammatory cells, and DAMPS from the innate immune system and PAMPS from fragments of viral, bacterial, or fungal elements add additional secondary BBB injury [44,49]. Both mechanisms alter the normal perfusion dynamics. In a recent study we conducted, evaluating college athletes post-mild acute traumatic brain injury using the ASL MRI technique (discussed below), we were able to identify reduced capillary mean transit time (cMTT)/glymphatic clearance rate acutely and demonstrated return to normal clearance at recovery [50]. Thus, a restoration pathway exists in youth, at least with minor TBI, but becomes dysfunctional with repetitive trauma leading to CTE and in ”normal” aging adults [26,35]. With future clearer understanding of the repair mechanisms in youth, restorative treatments could potentially be developed and applied to advanced-age-related BBB leakiness.

With age, low-grade inflammatory insults are leaked via subsequent glial upregulation of inflammatory interleukin cytokines (IL1b, IL6, IL-12, IL-23, and TNF-α), monocyte/lymphocyte-attracting chemokines (CCL2-CCL4-CCL7), and leukocyte-attracting CXCL10. Microglial cells convert to Type 1 with the expression of complement components C1q and C3a with inflammation or the presence of PAMPS from prior/dormant viral encephalitis (HSV-1, HHV6, HHV7, and EB virus) or chronic infection (periodontal disease, chlamydia, and fungi) [51,52,53,54,55,56,57,58].

**Figure 4:**
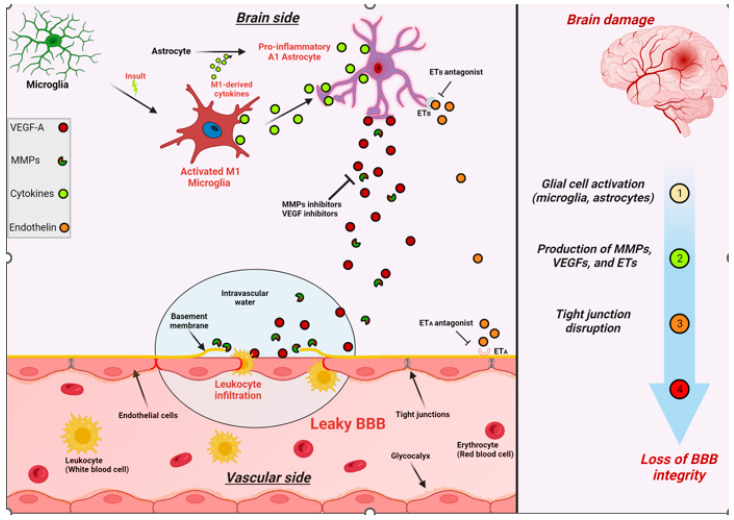
Schematic illustration summarizing the effects of brain damage on BBB integrity. The production and activation of MMPs, VEGFs, and ETs are upregulated in various brain cells following brain damage. These factors can then impair the viability and integrity of the BBB by negatively impacting the tight junctions between adjacent endothelial cells.

Additionally, diabetes, obesity, autoimmune inflammatory processes, whole-brain radiation treatments, tobacco abuse, and trauma impact the BBB TJs [34,44,49]. The expression of the C3a fragment complements and upregulates the endothelial C3a receptor, which causes profound phenotypic endothelial cellular changes [56,58]. The endothelial cells transform into immune permissive/attractant cells with the retraction of the TJ proteins via cytoskeletal actin contraction, causing leaks as well as the expression of VCAM1, attracting CD8 cells to the cell surface and entry into the interstitium with inflammation [56]. Here, it should be noted that there is a substantial contribution to the upregulation of interstitial inflammation by complement prevalent in microglia and astrocytes [56,58]. If low-level leakiness is sustained, the resulting altered metabolic processes cause longer-term corruption of protein synthesis, degradation with accumulating misfolded toxic proteins, and microvasculopathy in the case of neurodegenerative diseases [26,33,34,35,36,48,59,60]. The rapidity of development and the severity of misfolded protein accumulation is enhanced by well-known genetic genotypes, most commonly APOE 4 carriers [3,4,5,8,9] (Figure 4).

The enterobiome is also a factor increasingly being investigated in the development of neurodegenerative disease with clear involvement in Parkinson’s disease [61,62,63]. Enterobiome production of α synuclein protein with ascent and brain stem incorporation via Vagal afferents is well documented [62]. Bacterial-derived Lipopolysaccharide (LPS) and High mobility group box 1 (HMGB1), a nonhistone nucleoprotein that normally has intracellular maintenance function, once excreted, becomes a potent proinflammatory substance once extracellular [64]. LPS is a classic PAMP and HMGB1 is a classic DAMP with resultant upregulation of inflammation and both have been shown to induce BBB leak [51,64].

**Figure 5:**
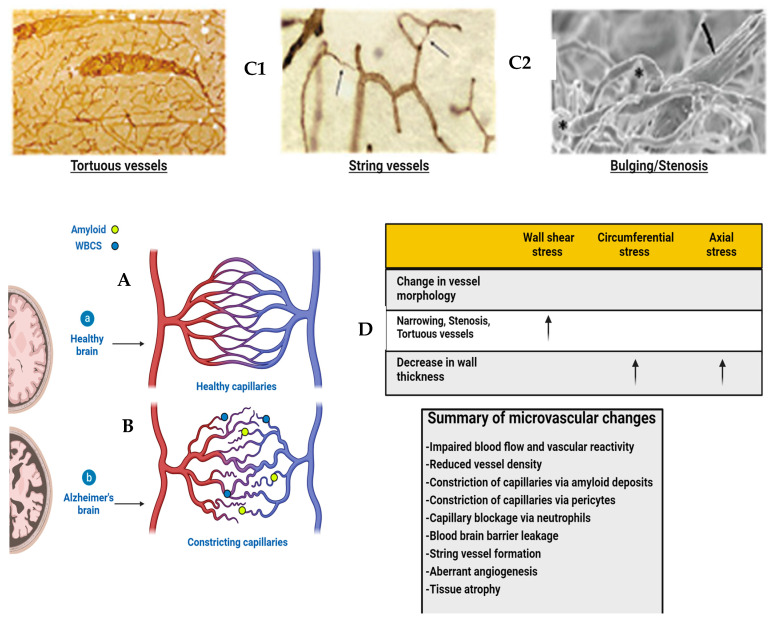
Diagram of healthy and Alzheimer’s brains’ vasculature. (**A**) Diagram of healthy brain vasculature. Penetrating arteries (red) and veins (blue) are connected via a capillary mesh (gray). Adapted from [69]. (**B**) Diagram of Alzheimer’s brain vasculature. Deposition of amyloid (yellow dots) in the vascular bed causes vascular dysfunction and loss in AD, with proliferation of vessels around missing vasculature (Adapted from [70]). String vessels (black) with no blood cells form as endothelial cells undergo apoptosis. Neutrophils (blue dots) block a small portion (~2%) of capillaries, reducing blood flow in the network. Penetrating vessels have a tortuous shape, with a tendency to decrease in diameter with aging in AD. (Adapted from [71]) Histopathology shows vascular abnormalities such as tortuous arterioles and string vessels (arrows) in the AD brain. Celloidin sections were stained for collagen. (**C1**,**C2**) Scanning electron microscopy of corrosion casts of the cerebral vasculature of an arcAbeta mouse revealed degenerated vessels (arrow). In addition, stenosis and bulging of the vessel wall are observed both on arteries and arterioles (asterisks). Scale bar 50 μm. Modified from [26]. (**D**) Table with effects of changes in vessel morphology on biomechanical properties. This article is licensed under the Creative Commons Attribution-Noncommercial-No Derivatives 4.0 International License (CC BY-NC-ND). Adapted from [48].

Vascular insult related to sustained hypertension causes vascular shear stress and damage to glycocalyx resulting in reduced heparin sulfate and proteoglycan content with a loss of antithrombotic and anti-inflammatory effects. The damage that ensues is produced by accumulating reactive oxygen species with the resultant upregulation of metalloproteases and damage to the BBB, as well as the upregulation of inflammatory cytokines [35,36,37,38,39,65]. If severe or sustained, each of these injuries causes structural alterations to the microvasculature with thinning, elongation, and increased tortuosity of the affected vessels due to the upregulation of metalloprotease enzymes related to increased oxidative stress and inflammatory changes [65]. The normal regional perfusion of the brain region is thus altered such that there is reduced blood flow, prolonged mean capillary transit time, and glymphatic flow impairment [66]. These same vascular changes develop in both head injury and chronic inflammatory-related BBB leaks in the absence of normal repair signaling/or pathways [12,67,68] (Figure 5).

The “normal” aging process Is associated with loss of pericytes, (etiology unknown) likely disrupting the integrity of the BBB with resulting leakiness [12,67,68]. But is their loss the consequence or cause of the BBB leak? There is also thickening of the endothelial basement membrane and alterations in constituent proteins along with the loss of pericytes and presence of reactive astrocytes and microglia, reducing neurovascular coupling [26,33,34,35,36,37]. The BBB leakiness goes undetected for years until there is a mild loss of cognitive abilities [60,68]. Progression to more severe dementia is not a certainty, with progression over 10 years in approximately 40% of patients. Modifiable risk factors for developing cognitive impairment include uncontrolled hypertension, obesity, poorly controlled diabetes, TBI, tobacco, and excess alcohol use are well recognized and listed with a level of evidence in the combined WHO and 2020 Lancet Commission Report [72,73].

Insulin resistance has also been identified, but whether it is a cause or result of endothelial cell dysfunction with decreased glut 1 transporter expression is unclear [8]. Those carrying one copy of APOE 4 have a 40% greater chance of developing AD dementia progression, and those with two copies have 10 times increased risk of early-age or late-age AD development, and this is currently non-modifiable [72,73]. In addition, the potential exists to therapeutically target the dysfunctional BBB by inhibiting the microglial and astrocyte conversion to their proinflammatory phenotype, inhibit the astrocyte-secreted inflammatory species and ROS-activated metalloprotease enzyme systems, and promote expression of the cadherin proteins at the BBB [74]. Management of early neurodegenerative disease development will require the repair of blood–brain barrier dysfunction. Before we tackle this, a more robust delineation of BBB repair mechanisms, operational in youth, is required, which can potentially be upregulated in age-related leakiness.

Direct intra-arterial vascular accumulation of βamyloid and resulting pro-hemorrhage and vasoocclusive effect has been addressed elsewhere and will not be addressed here [75,76].

This mounting evidence for BBB dysfunction being the nexus of neurodegenerative disease development can no longer be ignored. The co-occurrence of microvascular disease in addition to the interstitial metabolic inflammatory changes in most patients suggests they are two prongs of the same disease process and likely develop concurrently [43,45]. The accelerant factor of the ongoing accumulation of toxic misfolded proteins results in the progressive expansion of the BBB dysfunction and, ultimately, the loss of neurons and supporting structures with the resulting well-known cognitive impairments [48,60]. The pattern of involvement typically begins in the highly metabolically active hippocampal structures but with pertinent exceptions of clinical presentations such as in the Frontal temporal, Parietal, and, rarely, Occipital lobe [37].

The co-occurrence of both “small vessel disease” (by MRI) in the absence of large vessel atherosclerosis and associated neurodegeneration with the accumulation of βamyloid hpTau, as seen almost universally in neurodegenerative disease, strongly suggests the two processes are a spectrum of the same disease process [35,36]. The nexus is the loss of stability/integrity of the BBB (Figure 4). If this is so, then the physiological consequences of perfusion must be measurable in the early preclinical stages of a disease. This produces clinical challenges for early recognition and surveillance, and a major change in therapeutic focus to address potential means of restoring BBB integrity. The next section will discuss the early BBB leak and potential noninvasive means of detection. Without valid, safe methods of BBB leak identification, outcomes from future early intervention trials are impossible in a reasonable time frame, as cognitive decline may take years to develop.

## 2. Altered Fluid Dynamic

Hemodynamic flow is altered *regionally* post-BBB leak [77,78]. The mechanism is related to the microvascular/glymphatic flow of anatomical changes alluded to above. There is reduced blood volume in the affected areas, but in the absence of concomitant large or medium-sized arterial disease, the mean arterial transit time (aMTT) remains normal [59,66]. Just as a leaky garden hose increases the transit time of water flow out when compared to an intact one, so does a leaky BBB prolong regional mean capillary transit times (cMTT) [59,66]. In addition, the disruption in the BBB reduces signaling from astrocytes to endothelial cells of its metabolic needs and, thus, reduces the expression of required transporters and vesicular pathways [59,66]. This cascade is the result of glycocalyx and pericyte loss, basement membrane damage, and metabolic disruption caused by an extravascular leak of restricted substances [34]. The basement membrane damage shuts down the associated glymphatic system due to the retraction of astrocyte aquaporin channels from end feet to soma. Put simply, with BBB dysfunction, the reduced volume of fluid is slow to arrive and cannot get out. The pathological sequelae allow for persistent smoldering of inflammatory processes downstream that, over time, result in well-recognized misfolded protein accumulations associated with neurodegenerative disease [3,4,5,6,7,8,35].

## 3. Early Identification of BBB Dysfunction

**Figure 6:**
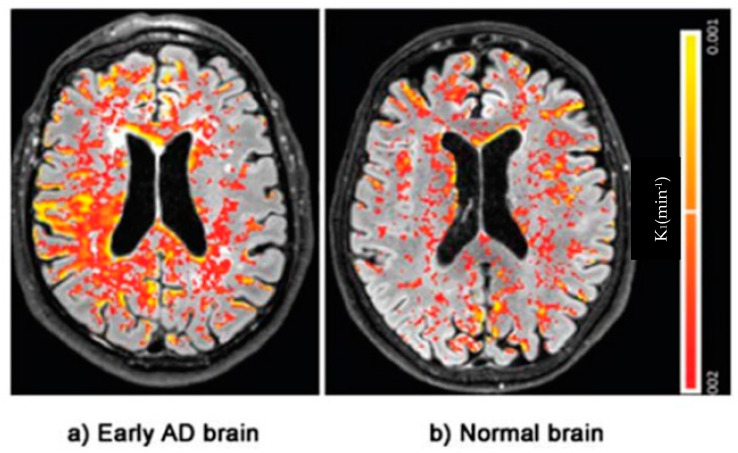
DCE Magnetic resonance brain image of an Alzheimer’s patient with color-coded (Red–Yellow) blood–brain barrier leakage. Note the greater extent of leakage in AD. Adapted from Evidence of BBB damage in the AD brain: (**a**) extensive leakage of gadobutrol (an MRI contrasting agent) through a damaged BBB in brains of patients with early signs of AD; (**b**) less extensive leakage of the agent in brains of normal patients. Adapted with permission from [77], published by the Radiological Society of North America, Inc., 2016.

Redirecting clinical research efforts to remedy the preclinical BBB dysfunction will require validated outcome measures to assess efficacy. A combination of imaging techniques addressing the leak or resulting physiological consequences coupled with serological as opposed to CSF evidence of brain injury would be ideal. Paradigms testing cognitive dysfunction/function are abnormal late in the disease course at a time when treatment is likely futile. So, in order to demonstrate the positive effect of future early treatments, outcome measures addressing the effects of BBB leak must be validated. To identify early BBB disruption, an imaging study should either survey the vascular leak or reflect the altered physiology present. The interstitial leak of fluid can be directly imaged using dynamic contrast enhancement (DCE) MRI imaging. BBB leak has been identified in aging adults without cognitive impairment localized initially in the hippocampal region by DCE-MRI [37,68,79]. This technique measures gadolinium contrast extravasation into the brain parenchyma relative to the arterial concentration in specific brain regions. Most notably, the Hippocampal region and sub-regions within were found to demonstrate a quantifiable contrast leak [37] (Figure 6). This technique uses the Patlak analysis to identify the subtle leak of contrast. The technique requires gadolinium contrast injection currently with scan times of 25–30 min. Faster scan methods are under development. Limitations of this technique are the long scan times per sequence and required gadolinium making multiple follow-up studies problematic.

Another approach for the early identification of BBB leak in the preclinical and early clinical state of disease leverages the regional hemodynamic changes resulting from disruptions in normal microvascular anatomy and leaky vessels [48,66,68]. The disruption in neurodegenerative disease causes reduced cerebral vascular flow volume but does not result in changes in the arterial mean transit time (aMTT) [66,67,68]. The mean capillary mean transit time (cMTT), however, is prolonged and glymphatic outflow is reduced. Noninvasive 3D ASL MRI indirectly assesses the clearance of labeled protons in the late stage of perfusion by assessing the perfusion signal at multiple time points post-labeling (PLD) and, using linear analysis, measures the slope of clearance [80]. This method identifies perfusion alterations resulting from BBB leak before the accumulation of β Amyloid or hpTau. In our effort to develop a usable MRI technique for identifying perfusion changes in AD, three specific goals had to be met. The first was the availability of the technique to any community hospital with a 3T MRI scanner. The second was it had to be time-efficient and noninvasive since multiple scans would likely be required over time; hence, a non-contrast study was imperative. The third was cost efficiency. The medical system here or elsewhere cannot absorb high-volume, high-cost diagnostic studies in a pervasive disease of this sort.

**Figure 7:**
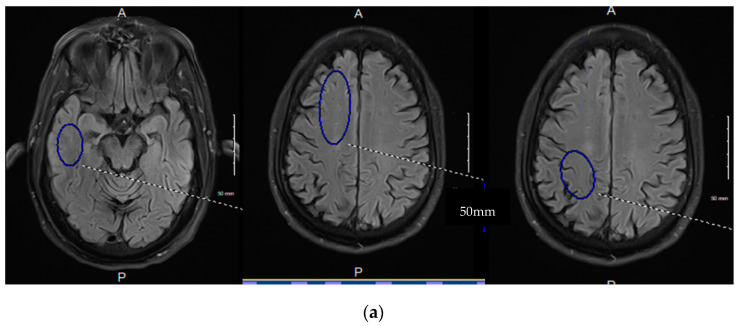

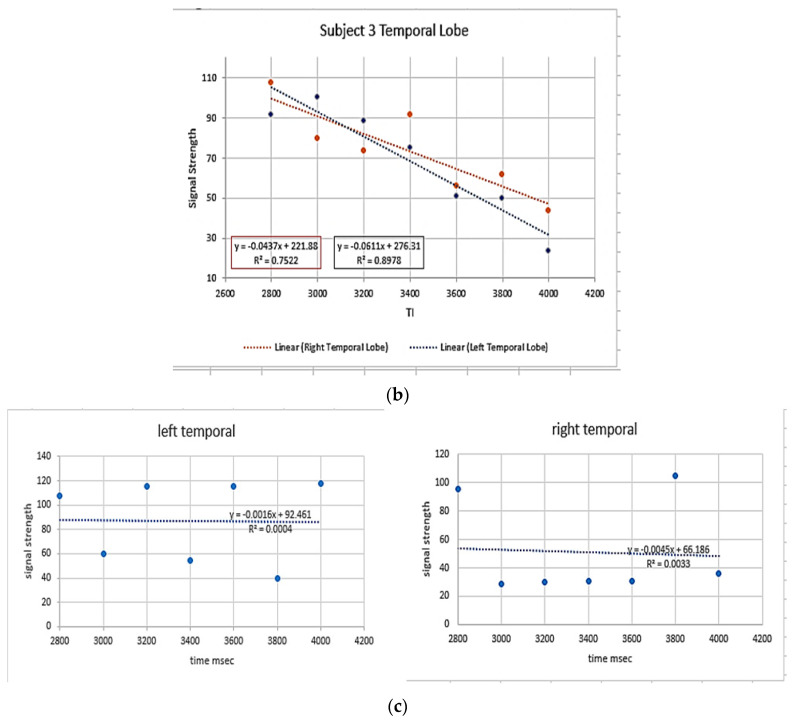
(**a**–**c**) 3D ASL glymphatic clearance rate in MCI and AD. (**a**). The three images denote the region of interest and size recorded for each region held constant bilaterally and across all subjects. The 4 mm slice angle and level for each region were also held constant. The lower images demonstrate the linear analysis of the 7 data points in an MCI patient with normal GCr (**b**). compared with an AD subject with reduced GCr (**c**). GCr (glymphatic clearance rate).

This technique uses 3D arterial spin labeling capturing the signal averages at multiple time points late in the perfusion cycle. The technique subtracts the background signal leaving a residual labeled proton signal from perfusion. The paradigm records the residual signal after time delays post-labeling beginning at 2800 ms through 4000 ms at 200 ms intervals (7 data points) [80]. By doing so, we can legitimately use linear analysis for the “glymphatic clearance rate” as the correlation with the T1 decay times of the constituent components in the late phase of perfusion have correlation coefficients of over 96% [80]. Signal averages in six regions of interest, bitemporal, bifrontal, and biparietal are investigated using standard volume and anatomical locations [80] Figure 7a–c.

The resultant signal averages are then easily transferred to a spreadsheet for graphical analysis, with the slope being the glymphatic clearance rate. This technique fulfills our major requirements in that it is non-invasive, time-efficient (approximately 2 min per ASL sequence and with T2 flair and susceptibility imaging resulting in a 20-min total scan time), and cost-efficient (an approximately $300 Medicare reimbursement Quote from the Medicare website). In a small case series report (that COVID-19 interrupted), we were able to demonstrate progression in one subject with MCI to precursor MRI changes of more advanced dementia prior to significant MMSE changes, a second patient with stable MRI changes and no cognitive decline, and a third patient with typical AD dementia and associated ASL MRI changes [81]. The technique is still in the development stage but will be enhanced with the general release of 3D ASL acquiring multiple PLD determinations following a single spin labeling. Also, the segmentation programs should speed up the cumbersome data analysis by ROI. The limitation of ASL is that a low level of signal is obtained in the late phases of perfusion. By using multiple PLDs and large ROIs, the artifact is reduced.

It is important to note that both directly imaging BBB leak of contrast and the determination of clearance by ASL are not specific for neurodegenerative disease but can be found in acute head injury, CNS infection, brain tumor, and acute stroke or, in other words, in any condition that alters the integrity of the BBB. That said, given the clinical suspicion of early disease or a high-risk profile for developing neurodegenerative disease, surveillance in the future when effective means of aborting the BBB damage is available will be necessary.

Serological markers of NVU injury would enhance the sensitivity of the testing. GFAP is a sensitive measure of β Amyloid accumulation and is thus helpful in identifying mid-stage disease [14,15]. Small acidic calcium-binding protein S100β is a sensitive but nonspecific marker of BBB leak [14]. The two markers may serve to corroborate imaging findings in the early phase of the disease (S100β). The GFAP concentration, if elevated, would suggest disease progression along with imaging study changes. Hence, concomitant use would provide a convenient and sensitive noninvasive means of diagnosis and surveillance of the initial stages of neurodegenerative disease.

From a clinician/patient standpoint, sensitive early detection using noninvasive MRI technology with a serological marker of brain injury to establish the preclinical diagnosis would be a welcome advance, particularly when effective early treatment exists.

## 4. Current Methods of Risk Reduction

**Figure 8:**
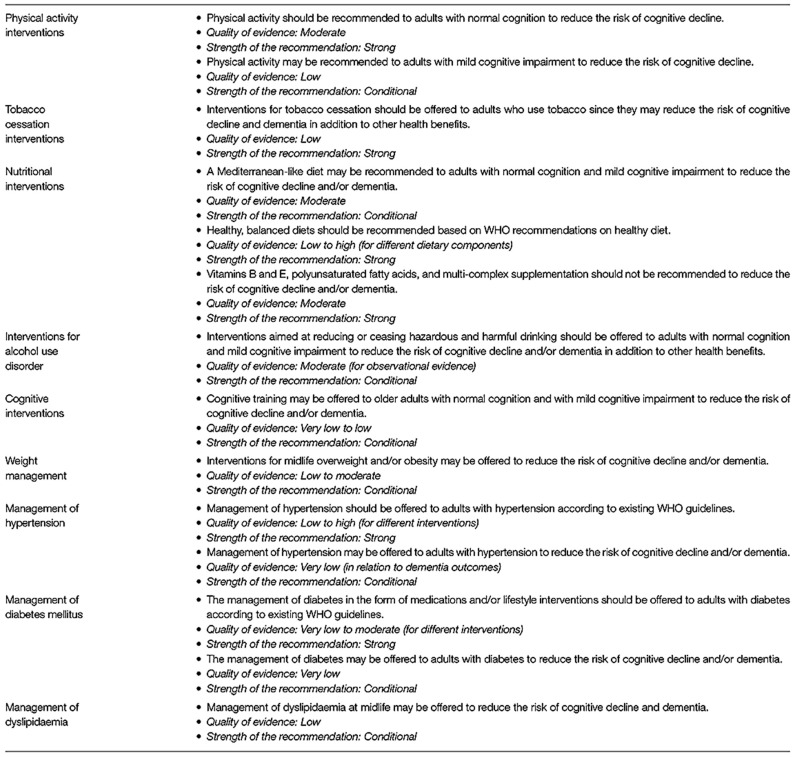
WHO recommendations for dementia prevention with quality and strength of medical evidence determinations. Adapted from [68].

There is unambiguous evidence that smoking cessation and successfully managing diabetes, hyperlipidemia, hypertension, and obesity decrease the risk of late-life dementia [82]. Likewise, treating hearing impairment in middle age and a reduction in alcohol use to no more than 21 U of alcohol (1 unit =10 cc alcohol) are also factors [73]. An education level less than high school (12 years of formal education) is associated with higher dementia risk [68]. Increasing aerobic exercise to 45–60 min per session at any frequency reduced the incidence of dementia [72,73]. Food supplements are of no value in preventing dementia, but a healthy diet (Mediterranean diet) is indirectly beneficial by reducing cardiovascular risk. Social isolation and depression are also risk factors, with reduced (<4 h/night) or excessive sleep (>10 h sleep) as an additional risk (Figure 8). Formal WHO 9-point risk factors with 3 additional risk factors of excessive alcohol consumption, TBI, and possibly air pollution were added by the Lancet study group. The clearance of antigenic senescent cells (“Zombie cells”) accumulating with age is also a promising future target for pharmacological clearance. A phase 1 trial using Senolytic therapy has been put forward as a potential treatment for alleviating cognitive deficits in AD [83,84].

**Figure 9:**
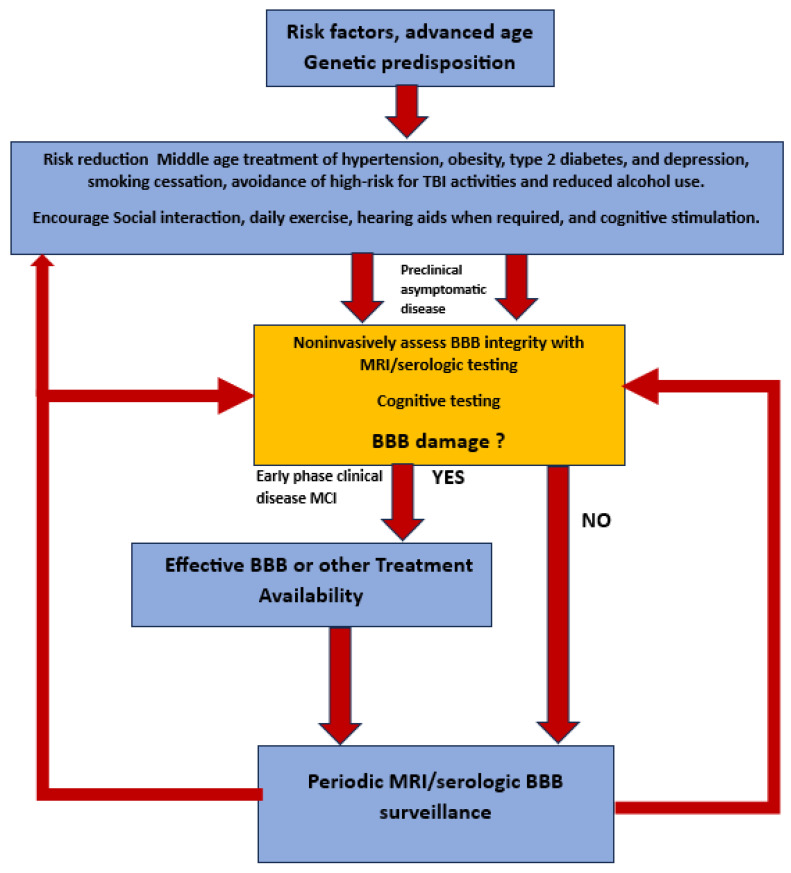
Flow diagram of potential future approach to preclinical diagnosis and surveillance of high-risk individuals for developing AD once effective treatment paradigms are available.

To that end, the early identification of patients at risk of chronic poorly controlled diabetes, hypertension, chronic infection, identifiable enterobiome factors, genetic predisposition, and multiple head traumas will require surveillance for BBB leak at an early age and on a periodic basis. Once treatment is initiated, continued surveillance will be required to ensure the reversal/cessation of BBB leakiness and cognitive change (Figure 9). Cheap, noninvasive, time-efficient, and validated diagnostic testing will be mandatory. In short, once early successful treatment is available, a major shift in clinical thinking must take place specifically, we will need to *find it before it finds us*!

## 5. Conclusions—Where Do We Go from Here?

An innovative approach to early diagnosis and preemptive treatment of neurodegenerative disease based on the recognition and mitigation of blood–brain barrier dysfunction, the nexus of these diseases, must be addressed. AD is highly likely to be a spectrum disease causing both microvasculopathy and altered protein synthesis and degradation with accumulating toxic misfolded proteins. The latter events accelerate the BBB dysfunction, producing a vicious cycle with a loss of neural components and, ultimately, cognitive decline. The disappointing effect of removing accumulating misfolded proteins (β amyloid) demonstrates the need to intervene early but address a different target, specifically repairing the BBB leakiness.

Step 1: For now, reducing the peripheral inflammatory load (multiple sources) and controlling vascular/glycocalyx shear forces from uncontrolled hypertension are essential, thereby reducing the burden on the age-related leaking BBB. Middle-age treatment of hypertension, obesity, type 2 diabetes, and depression, smoking cessation, avoidance of high-risk TBI activities, and reduced alcohol use should be carefully explained to patients in terms of reducing the risk of long-term cognitive loss. Social interaction, exercise, hearing aids when required, and cognitive stimulation are all positive influences on maintaining intellectual function. Developing reliable multimodality screening methods for early vascular damage, which correlate with early BBB injury, that are low-cost, dependable, and adoptable worldwide is the logical next step toward early intervention when the latter is available.

Step 2 is restoring the body’s natural BBB repair mechanism that is slowly lost with the aging process. To move in that direction requires firmer knowledge of the repair signaling pathways that are present in youth but begin to slowly fail with age. Once this is clarified, the target/s for therapeutic intervention can be exploited. Validated, non-invasive, economically sound MRI/serologic methods will need to be in place to both make a pre-clinical diagnosis and judge the efficacy of future treatment outcomes.

## Data Availability

All data from the previously published clinical studies performed by the author are available upon request as stated in those publications.
